# Upregulating the mevalonate pathway and repressing sterol synthesis in *Saccharomyces cerevisiae* enhances the production of triterpenes

**DOI:** 10.1007/s00253-018-9154-7

**Published:** 2018-06-15

**Authors:** Jan Niklas Bröker, Boje Müller, Nicole van Deenen, Dirk Prüfer, Christian Schulze Gronover

**Affiliations:** 1 Institut für Biologie und Biotechnologie der Pflanzen, Westfälische Wilhelms-Universität Münster, Schlossplatz 8, 48143 Münster, Germany; 2 Fraunhofer Institut für Molekularbiologie und Angewandte Oekologie, Schlossplatz 8, 48143 Münster, Germany

**Keywords:** Metabolic engineering, MVA pathway, Sterol biosynthesis, tHMGR, Pentacyclic triterpenes, *Saccharomyces cerevisiae*

## Abstract

Pentacyclic triterpenes are diverse plant secondary metabolites derived from the mevalonate (MVA) pathway. Many of these molecules are potentially valuable, particularly as pharmaceuticals, and research has focused on their production in simpler and more amenable heterologous systems such as the yeast *Saccharomyces cerevisiae*. We have developed a new heterologous platform for the production of pentacyclic triterpenes in *S. cerevisiae* based on a combinatorial engineering strategy involving the overexpression of MVA pathway genes, the knockout of negative regulators, and the suppression of a competing pathway. Accordingly, we overexpressed *S. cerevisiae ERG13*, encoding 3-hydroxy-3-methylglutaryl-coenzyme A (HMG-CoA) synthase, and a truncated and deregulated variant of the rate-limiting enzyme HMG-CoA reductase 1 (tHMGR). In the same engineering step, we deleted the *ROX1* gene, encoding a negative regulator of the MVA pathway and sterol biosynthesis, resulting in a push-and-pull strategy to enhance metabolic flux through the system. In a second step, we redirected this enhanced metabolic flux from late sterol biosynthesis to the production of 2,3-oxidosqualene, the direct precursor of pentacyclic triterpenes. In yeast cells transformed with a newly isolated sequence encoding lupeol synthase from the Russian dandelion (*Taraxacum koksaghyz*), we increased the yield of pentacyclic triterpenes by 127-fold and detected not only high levels of lupeol but also a second valuable pentacyclic triterpene product, β-amyrin.

## Introduction

**Fig. 1:**
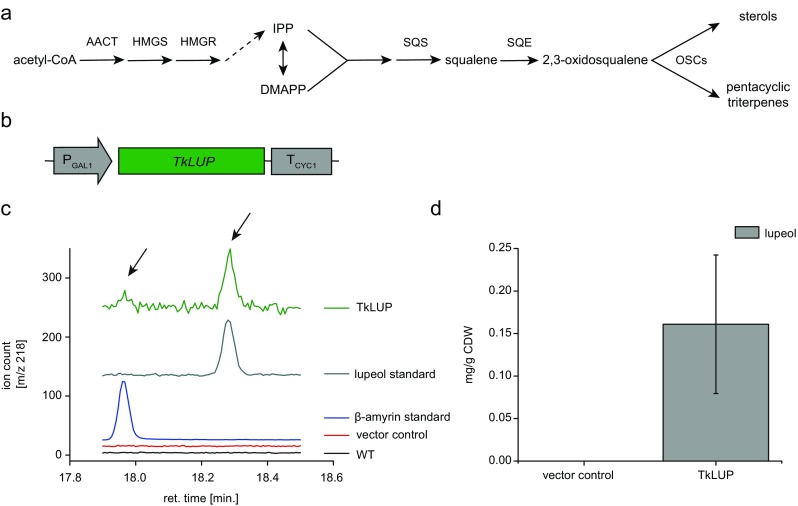
Triterpene accumulation in the yeast *S. cerevisiae* expressing TkLUP. **a** Schematic representation of the MVA pathway leading to the synthesis of sterols and pentacyclic triterpenes via oxidosqualene cyclases (OSCs). Dashed arrows represent multiple enzymatic reactions. AACT = acetyl-CoA C-acetyltransferase; DMAPP = dimethylallyl pyrophosphate; IPP = isopentenyl diphosphate; HMGS = 3-hydroxy-3-methylglutaryl-coenzyme A (HMG-CoA) synthase; HMGR = HMG-CoA reductase; SQS = squalene synthase; SQE = squalene epoxidase. **b** Schematic representation of the *TkLUP* coding sequence under the control of the *GAL1* promoter (P_GAL1_) and *CYC1* terminator (T_CYC1_). **c** Yeast cells carrying the *TkLUP* coding sequence showed two additional peaks in the GC-MS spectrum (*m*/*z* = 218, arrows), probably representing β-amyrin (retention time = 17.95 min) and lupeol (retention time = 18.25 min) because they match the corresponding standards. **d** Yeasts carrying the *TkLUP* coding sequence accumulated 0.16 mg/g CDW of the putative lupeol but the quantification of the β-amyrin peak was not possible. Wild-type (WT) and pAG424_P_GAL1_-*ccdb* vector control CEN.PK2-1C cells served as controls. The standard deviation was calculated from *n* = 3 individual transformants; CDW = cell dry weight

Isoprenoids are a diverse group of natural compounds found in all living organisms, with at least 50,000 different structures already reported (Hemmerlin et al. 2012; Liao et al. 2016). In plants, these products are derived from the plastididial 2C-methyl-d-erythritol 4-phosphate (MEP) and the cytosolic mevalonate (MVA) pathway. In the latter, acetyl-CoA is converted to the isoprenoid precursor isopentenyl diphosphate (IPP) via six enzymatic steps. Two important MVA pathway enzymes are the sequentially acting 3-hydroxy-3-methylglutaryl-coenzyme A (HMG-CoA) synthase (HMGS) and HMG-CoA reductase (HMGR), the latter representing the rate-limiting step (Demierre et al. 2005). IPP is isomerized to form dimethylallyl pyrophosphate (DMAPP), and together, IPP and DMAPP can act as substrates for various isoprenoid-derived pathways. For example, two molecules of IPP and one of DMAPP can be converted into farnesyl pyrophosphate (FPP) which in turn can be converted into squalene by squalene synthase (SQS). The oxidized form of squalene (2,3-oxidosqualene) is a precursor for the synthesis of sterols (leading to the production of lanosterol in fungi and animals, or cycloartenol in plants) and also pentacyclic triterpenes (Fig. 1a), the latter involving various oxidosqualene cyclases (OSCs) such as lupeol synthase in the dandelion *Taraxacum officinale* and β-amyrin synthase in the wormwood plant *Artemisia annua* (Shibuya et al. 1999; Kirby et al. 2008). The products of these enzymes can be further metabolized by acylation or oxidation. The efficient triterpene oxidation of, e.g., lupeol to betulin and betulinic acid by P450 enzymes could be demonstrated in yeast (Zhou et al. 2016). FPP is also a precursor for the synthesis of sesquiterpenes, e.g., farnesene or amorpha-4,11-diene, a precursor of the anti-malarial drug artemisinin (Martin et al. 2003).

The value of isoprenoids, particularly as pharmaceuticals, has prompted the development of heterologous production systems including the yeast *Saccharomyces cerevisiae* (reviewed by Liao et al. 2016 and Vickers et al. 2017)*.* The potential of the yeast MVA pathway for the production of isoprenoids was first demonstrated by overexpressing the catalytic domain of HMGR (tHMGR), which increased the yield of squalene (Donald et al. 1997). The consequences of overexpressing other MVA pathway genes were determined by combinatorial library screening for the overexpression of *ERG10* (acetoacetyl CoA thiolase; AACT), *ERG13* (HMGS), and *ERG12* (mevalonate kinase) which enhanced the production of amorpha-4,11-diene (Yuan and Ching 2014). The MVA pathway has also been targeted using the CRISPR/Cas9 system, revealing loci that trigger the accumulation of mevalonate and triterpenes when knocked out (Jakočiūnas et al. 2015; Arendt et al. 2017). The targets included *ROX1*, encoding a transcriptional regulator that inhibits genes involved in the MVA pathway and sterol biosynthesis (Henry et al. 2002; Montañés et al. 2011; Özaydin et al. 2013; Jakočiūnas et al. 2015). The insertion of regulated promoters into the yeast genome can also suppress genes involved in endogenous but competitive isoprenoid pathways to redirect the metabolic flux in a more precise and desirable manner. Accordingly, the methionine sensitive *MET3* promoter was used to downregulate the expression of the lanosterol synthase gene (*ERG7*), which represents the first committed step in the late sterol biosynthesis pathway, enhancing the production of β-amyrin (Kirby et al. 2008). A similar strategy was used to suppress the squalene synthase gene (*ERG9*), allowing the enhanced production of artemisinin (Ro et al. 2006; Westfall et al. 2012) until the authors changed their strategy and used the copper transporter 3 (*CTR3*) promoter, which allows transcriptional repression to be induced by adding CuSO_4_ to the medium, thus reducing the costs of industrial processes (Paddon et al. 2013).

Here, we developed a new yeast-based platform for the synthesis of triterpenes, combining the overexpression of the MVA pathway genes *ERG13* (HMGS) and a truncated version of *HMG1* (tHMGR), the disruption of the *ROX1* gene, and the copper-regulated repression of *ERG7* using the *CTR3* promoter. By implementing this platform, we were able to enhance the productivity of the MVA pathway and redirect metabolic flux from late sterol biosynthesis, resulting in a 127-fold increase in the yield of lupeol using lupeol synthase from the Russian dandelion *Taraxacum koksaghyz* (TkLUP) as a model enzyme.

## Materials and methods

### Cloning of constructs

The construct pAG424_P_GAL1_-TkLUP was prepared by amplifying the *TkLUP* coding sequence (GenBank MG646375) from *T. koksaghyz* cDNA using forward primer 5′-AAA GTC GAC TAA AAA AAT GTG GAA GCT GAA AAT AGC-3′ and reverse primer 5′-AAA CTC GAG ATA TAT TTT GAA CAA TAC GA-3′ (restriction sites are underlined). The PCR product was purified, digested, and inserted into pENTR3c (Invitrogen, Carlsbad, USA). The *TkLUP* coding sequence was then introduced into pAG424_P_GAL1_-ccdB (Alberti et al. 2007; Addgene, Cambridge, USA) by LR recombination.

The construct pESC-rox1-KlURA3_tHMGR/ERG13 was generated by amplifying the *ROX1* coding sequence from *S. cerevisiae* genomic DNA using forward primer 5′-AAA GCG GCC GCA TGA ATC CTA AAT CCT CTAC-3′ and reverse primer 5′-AAA GCG GCC GCT CAT TTC GGA GAA ACT AGG-3′ (restriction sites are underlined). Furthermore, pESC-URA (Agilent Technologies, Santa Clara, USA) was used as a template to amplify a pESC-URA vector backbone containing *Not*I restriction sites using forward primer 5′-AAA GCG GCC GCC CAG CTG CAT TAA TGA ATC G-3′ and reverse primer 5′-AAA GCG GCC GCG AAG TTC CTA TTC TCT AGA AA-3′ (restriction sites are underlined). After digestion with *Not*I, the restriction fragments were ligated to obtain pESC-rox1. This vector was digested with *Bgl*II and a synthetic DNA fragment (Invitrogen), consisting of a Kl*URA3* marker cassette (Gueldener et al. 2002), and an *Asi*SI/Nb.*Bsm*I uracil-specific excision reaction (USER) cassette (Hansen et al. 2011) was inserted to obtain pESC-rox1-KlURA3. In a parallel approach, the coding sequences of the truncated *HMG1* gene (*tHMGR*) and *ERG13* were amplified from *S. cerevisiae* genomic DNA using forward primer 5′-AAA GGA TCC AAA AAA ATG GTT TTA ACC AAT AAA AC-3′ and reverse primer 5′-AAA GTC GAC TTA GGA TTT AAT GCA GGT GAC-3′ for *tHMGR* and forward primer 5′-AAA GAA TTC AAA AAA ATG AAA CTC TCA ACT AAA CTT TG-3′ and reverse primer 5′-AAA GCG GCC GCT TAT TTT TTA ACA TCG TAA GAT C-3′ for *ERG13* (restriction sites are underlined). The PCR products were digested with *Bam*HI/*Sal*I and *Eco*RI/*Not*I, and ligated into pESC-URA to generate pESC-URA-tHMGR/ERG13. The *Not*I restriction site was removed using the QuikChange Lightning Site-Directed Mutagenesis Kit (Agilent Technologies) according to the manufacturer’s protocol. In the final step, the expression cassette, containing the *tHMGR* and *ERG13* coding sequences, as well as the bidirectional *GAL1*/*GAL10* promoter and the *ADH1*/*CYC1* terminators, was amplified using forward primer 5′-CGT GCG A**U**T CAG AGC GAC CTC ATG CTA TAC-3′ and reverse primer 5′-CAC GCG A**U**C TTC GAG CGT CCC AAA ACC-3′ (uracil base for USER cloning shown in bold) and was introduced into pESC-rox1-KlURA3 (digested with *Asi*SI) according to the USER protocol.

The construct pESC_P_ERG7__KlLEU2_P_CTR3__erg7 was generated in a three-step process. In the first step, an *ERG7* fragment was amplified from *S. cerevisiae* genomic DNA using forward primer 5′-*CAC ATT TAA GGG CTA TAC AAA G*AT GAC AGA ATT TTA TTC TGA CA-3′ and reverse primer 5′-AAA GCG GCC GCC CCA ATA AAC GTA AGA TTA CA-3′, and a *CTR3* promoter fragment was amplified using forward primer 5′-AAA GCG GCC GCC AGC TGA AGG ATC CGG TAT TCC AAT GAG AAT CGC-3′ and reverse primer 5′-*TGT CAG AAT AAA ATT CTG TCA T*CT TTG TAT AGC CCT TAA ATG T-3′ (italic letters indicate the overlapping region; restriction sites are underlined). The products were fused by overlapping PCR using the *CTR3* promoter forward primer and the *ERG7* reverse primer. The spliced product was digested with *Not*I and transferred to the pESC-URA vector linearized with the same enzyme to obtain pESC_P_CTR3__erg7. In the second step, the upstream *ERG7* promoter fragment was amplified from *S. cerevisiae* genomic DNA using forward primer 5′-AAA CAG CTG AAT CTG CTG CTA TTC GTG-3′ and reverse primer 5′-AAA GGA TCC CCT GCA GGT CCG CAG ATA TCA AAT CTA G-3′ and was transferred to the *Pvu*II/*Bam*HI sites of pESC_P_CTR3__erg7 to obtain pESC_P_ERG7__P_CTR3__erg7. In the final step, a synthetic DNA fragment (Invitrogen) containing a Kl*LEU2* auxotrophy cassette (Gueldener et al. 2002) was ligated at the *Sbf*I/*Bam*HI restriction sites to obtain pESC_P_ERG7__KlLEU2_P_CTR3__erg7.

The integrity of all constructs was verified by sequencing (Sanger et al. 1977) on an ABI PRISM 3100 Genetic Analyzer (Applied Biosystems, Foster City, USA). Yeast strain CEN.PK2-1C was obtained from EUROSCARF (Oberursel, Germany). Restriction enzymes were obtained from New England Biolabs GmbH (Frankfurt a.M., Germany).

### Strain construction and culture conditions

The *S. cerevisiae* strain CEN-PK2-1C was transformed using the lithium acetate method (Gietz and Schiestl 2007) with *URA3* (pESC-rox1-KlURA*3*_tHMGR/ERG13), *LEU2* (pESC_P_ERG7__KlLEU2_P_CTR3__erg7), and *TRP1* (pAG424_P_GAL1_-TkLUP) as selectable markers. For stable integration into the yeast genome, pESC-rox1-KlURA3_tHMGR/ERG13 and pESC_P_ERG7__KlLEU2_P_CTR3__erg7 were digested with *Not*I to remove the plasmid backbone. The yeast cells were plated on minimal synthetic defined (SD) medium (Clontech, Mountain View, USA) and incubated at 30 °C. Clones were checked for integrity by colony PCR using primers spanning both ends of the integrated construct if needed.

For the expression of galactose-inducible genes, a single colony was picked, inoculated into 5 ml SD medium, and cultivated overnight at 30 °C on a rolling platform. From this culture, 50 ml of fresh SD medium (containing 150 μM CuSO_4_ when repressing the expression of *ERG7*) was inoculated to a final cell density of 10^5^ cells/ml and incubated at 30 °C shaking at 140 rpm in a 250-ml Erlenmeyer flask. When the culture reached a cell density of 0.4 × 10^6^ cells/ml, the medium was changed to SD medium containing galactose instead of glucose to induce gene expression. The cells were grown to a density of 4 × 10^6^ cells/ml and harvested by centrifugation (10 min, 1000×*g*).

### Squalene and triterpene extraction and quantitation

Yeast metabolites were extracted as described by Rodriguez et al. (2014). Briefly, freeze-dried yeast cells were incubated at 80 °C in a water bath for 5 min after adding 1 ml 6% [*w*/*v*] KOH in methanol (Carl Roth, Karlsruhe, Germany) and 100 μg cholesterol as an internal standard (Sigma, St. Louis, USA) to each sample. To extract the metabolites from the methanol mixture, 1 ml of *n*-hexane (Carl Roth) was added. After vortexing, the upper phase was transferred to a new vial and the extraction was repeated two times using 500 μl *n*-hexane. The *n*-hexane of the pooled extracts was removed by evaporation. The samples were re-solubilized in 1 ml acetone (Carl Roth) and analyzed by gas chromatography mass spectrometry (GC-MS) using a GC-MS-QP 2010 Ultra (Shimadzu, Duisburg, Germany) equipped with a 30-m Rtx-5MS column. After a 1-min hold at 120 °C, the temperature was increased to 330 °C at 21 °C per min (pressure = 58.8 kPa) followed by a hold of 330 °C for 10 min. Different compounds were identified according to their ion mass/charge ratios (43, 55, 69, 95, 109, 189, 204, 207, 218, 271, 285, and 411 *m*/*z*) by peak integration using LabSolution software (Shimadzu) and matching to the National Institute of Standards and Technology library. The total ion current of the detected substances was normalized against the cholesterol internal standard and the dry weight of the sample. The statistical significance of the results was confirmed using a two-sample *t* test at *p* < 0.05.

## Results

### Identification of TkLUP as a lupeol synthase from *T. koksaghyz*

We chose a lupeol synthase from the rubber-producing dandelion *T. koksaghyz* as a model enzyme for the new yeast platform. Using primers designed for the amplification of *TRX*, a lupeol synthase gene from *T. officinale* (Shibuya et al. 1999), we were able to amplify a 2277-bp open reading frame from *T. koksaghyz* cDNA encoding a polypeptide of 759 amino acids, showing 99.3% sequence identity to the *T. officinale* lupeol synthase. For expression in the wild-type (WT) *S. cerevisiae* strain CEN.PK2-1C, we inserted the sequence into pAG424_P_GAL1_-*ccdB*, which allows expression under the control of the *GAL1* promoter (Fig. 1 b). The empty vector pAG424P_GAL1__*ccdB* served as the vector control in the expression experiments. After cultivation and triterpene extraction, GC-MS analysis revealed two additional peaks (*m*/*z* = 218) in the extracts from three independent transformants expressing the lupeol synthase sequence, but these peaks were not observed in the WT or vector control samples (Fig. 1c). The retention times matched those of the β-amyrin and lupeol standards, indicating that the peaks reflected the accumulation of trace amounts of β-amyrin and ~ 0.16 mg/g cell dry weight (CDW) of lupeol (Fig. 1d). These data strongly supported the annotation of the new *T. koksaghyz* sequence as lupeol synthase (TkLUP).

### The deletion of *ROX1* and the overexpression of *tHMGR* and *ERG13* enhance the accumulation of lupeol by 16.5-fold

**Fig. 2:**
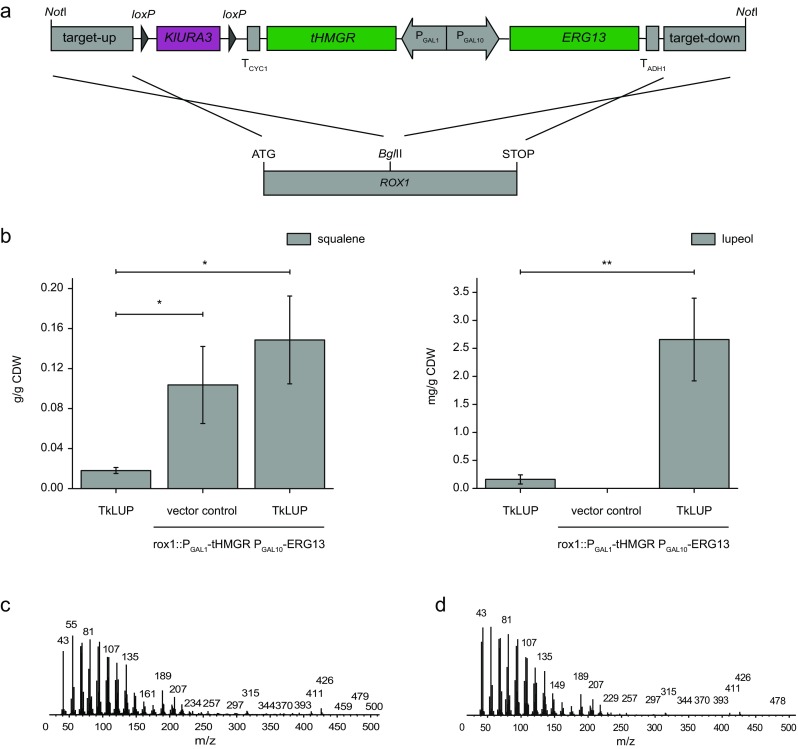
Accumulation of squalene and lupeol after the deletion of *ROX1* and the overexpression of MVA pathway genes. **a** Schematic representation of the construct for the deletion of *ROX1* and the overexpression of *tHMGR* and *ERG13*. The *tHMGR* and *ERG13* coding sequences were placed under the control of a bidirectional *GAL1*/*GAL10* promoter (P_GAL1_; P_GAL10_). Kl*URA3* was used to complement the uracil auxotrophy during the integration of the construct into the yeast genome. The locus for integration was defined by sequences flanking the construct (target-up; target-down) that were homologous to the *ROX1* target site. Transformation of the *Not*I-linearized construct led to the knockout of *ROX1* by homologous recombination. **b** Yeast strains carrying the integrated construct in addition to the *TkLUP* coding sequence (rox1::P_GAL1_-tHMGR P_GAL10_-ERG13 TkLUP) accumulated higher levels of the lupeol precursor squalene in contrast to cells carrying only the *TkLUP* sequence (*p* = 0.0137). Cells containing the empty vector pAG424_P_GALl__*ccdB* served as controls (rox1::P_GAL1_-tHMGR P_GAL10_-ERG13 vector control). The deletion of *ROX1* and the overexpression of *tHMGR* and *ERG13* resulted in a 16.5-fold increase in the lupeol content (*p* = 0.00893). **c** Mass spectrum of the designated lupeol peak. **d** Mass spectrum of the measured external lupeol standard. Standard deviation was calculated from *n* = 3 individual transformants. CDW = cell dry weight; one asterisk = *p* ≤ 0.05; two asterisks = *p* ≤ 0.01

To enhance the production of lupeol, we overexpressed two yeast MVA pathway genes that have previously been shown to improve the yield of isoprenoids in heterologous yeast systems (Kirby et al. 2008; Asadollahi et al. 2010; Paddon et al. 2013). HMGR (*HMG1*) catalyzes the rate-limiting step of the pathway and is subject to strict feedback control, so we overexpressed a truncated form of the enzyme (tHMGR) which no longer responds to feedback inhibition by enzyme degradation due to a missing ubiquitination signal (DeBose-Boyd 2008). We also overexpressed HMGS (*ERG13*) which promotes isoprenoid biosynthesis by supplying the substrate for HMGR (Yuan and Ching 2014). Finally, we knocked out the *ROX1* gene, which encodes a negative regulator of the MVA pathway and late sterol biosynthesis (Henry et al. 2002; Montañés et al. 2011; Özaydin et al. 2013; Jakočiūnas et al. 2015). We expressed the truncated *HMG1* gene and *EGR13* under the control of the bidirectional *GAL1*/*GAL10* promoter and integrated the entire overexpression cassette into the *ROX1* locus to concurrently knock out this gene (Fig. 2a). As expected, the overexpression of tHMGR and HMGS and the knockout of *ROX1* in yeast cells already expressing TkLUP resulted in a significant 8.2-fold increase in the squalene content as well as a 16.5-fold higher yield of lupeol compared to cells carrying the *TkLUP* sequence alone (Fig. 2b). There was also a 3.6-fold increase in the abundance of lanosterol (Table 1).

**Table 1:** Metabolite levels in wild-type (WT) and engineered yeast strains in g/g CDW quantified by GC-MS.

	Squalene	2,3-Oxidosqualene	Lanosterol	Ergosterol	Lupeol
WT	0.0248 (± 0.0078)	n.d.	0.01162 (± 0.00567)	0.0473 (± 0.01844)	n.d.
Vector control	0.0168 (± 0.0020)	n.d.	0.009172 (± 0.00203)	0.0332 (± 0.0061)	n.d.
TkLUP	0.0181 (± 0.0030)	n.d.	0.00803 (± 0.00148)	0.0289 (± 0.0042)	0.00016 (± 0.00008)
rox1::P_GAL1_-tHMGR P_GAL10_-ERG13	0.1539 (± 0.0126)	n.d.	0.03614 (± 0.01597)	0.0404 (± 0.0038)	n.d.
rox1::P_GAL1_-tHMGR P_GAL10_ ERG13 vector control	0.1037 (± 0.0385)	n.d.	0.03089 (± 0.01019)	0.0271 (± 0.0061)	n.d.
rox1::P_GAL1_-tHMGR P_GAL10_-ERG13 TkLUP	0.1486 (± 0.0439)	n.d.	0.02860 (± 0.00954)	0.0417 (± 0.0092)	0.00266 (± 0.00074)
rox1::P_GAL1_-tHMGR P_GAL10_-ERG13 P_ERG7_Δ::P_CTR3_ 0 μM CuSO_4_	0.1018 (± 0.0081)	0.0418 (± 0.0191)	0.10514 (± 0.03471)	0.0416 (± 0.0057)	n.d.
rox1::P_GAL1_-tHMGR P_GAL10_-ERG13 P_ERG7_Δ::P_CTR3_ 150 μM CuSO_4_	0.0442 (± 0.0018)	0.1974 (± 0.0345)	0.00645 (± 0.00290)	0.01765 (± 0.0054)	n.d.
rox1::P_GAL1_-tHMGR P_GAL10_-ERG13 P_ERG7_Δ::P_CTR3_ 375 μM CuSO4	0.0194 (± 0.0031)	0.2996 (± 0.0867)	0.00098 (± 0.00018)	0.0027 (± 0.0003)	n.d.
rox1::P_GAL1_-tHMGR P_GAL10_-ERG13 P_ERG7_Δ::P_CTR3_ vector control 150 μM CuSO_4_	0.0152 (± 0.0009)	0.0824 (± 0.0011)	0.00330 (± 0.00012)	0.0053 (± 0.0002)	n.d.
rox1::P_GAL1_-tHMGR P_GAL10_-ERG13 P_ERG7_Δ::P_CTR3_ TkLUP 150 μM CuSO_4_	0.057 (± 0.0097)	0.1443 (± 0.0479)	0.00463 (± 0.00040)	0.0107 (± 0.0017)	0.02032 (± 0.00472)

g/g CDW (± standard deviation); standard deviation was calculated from *n* = 3 individual transformants using Student’s *t* test

*n.d.* not detectable, *CDW* cell dry weight

The transformed yeast cells grew at a slower rate, as the time to reach the cell density for harvesting extended from 17 to 18.5 h, perhaps reflecting the toxicity of the higher squalene content (Donald et al. 1997; Asadollahi et al. 2010). However, this engineering step allowed us to compare the putative lupeol peak (Fig. 2c) with that of the lupeol standard (Fig. 2d). The comparable masses of the peaks supported our annotation of the TkLUP sequence. The putative β-amyrin peak was still too weak for detailed mass analysis.

### *ERG7* repression using the *CTR3* promoter causes 2,3-oxidosqualene to accumulate and achieves a further 7.6-fold increase in the lupeol content

The engineering steps outlined above resulted in the accumulation of squalene, but not of 2,3-oxidosqualene (the substrate of the TkLUP). This indicates that squalene synthase (ERG9) may have a higher metabolic capacity than squalene epoxidase (ERG1) as previously suggested by Asadollahi et al. (2010), perhaps exacerbated by the rapid conversion of the limited 2,3-oxidosqualene pool by TkLUP itself. Alternatively, competition from the endogenous late sterol biosynthesis pathway could draw flux away from the pentacyclic triterpenes. Yeast cells overexpressing *ERG1* accumulate 2,3-oxidosqualene but not downstream pentacyclic triterpenes (Veen et al. 2003), suggesting that competition from the endogenous late sterol biosynthesis pathway is primarily responsible for the low pentacyclic triterpene content in our cells. We therefore set out to suppress the expression of *ERG7* (lanosterol synthase) representing the first committed step in the late sterol biosynthesis pathway.

**Fig. 3:**
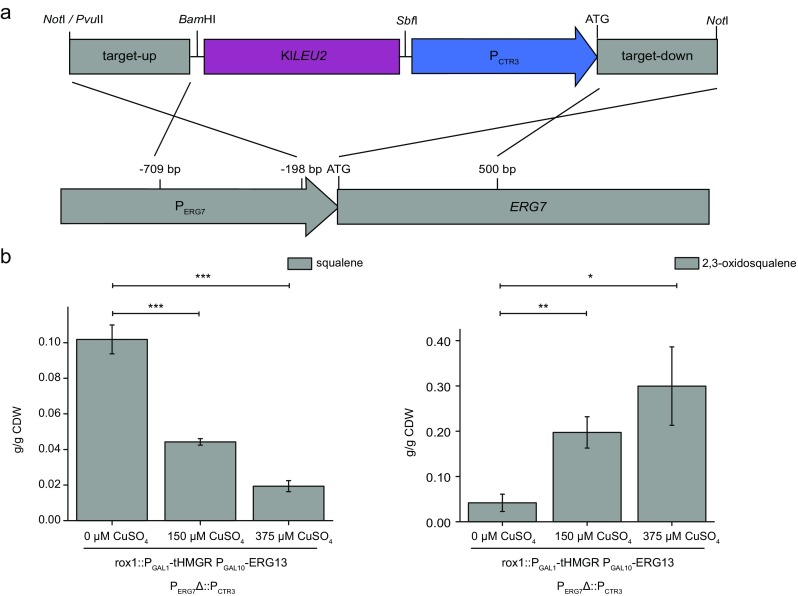
Repression of *ERG7* leads to the accumulation of 2,3-oxidosqualene. **a** Schematic representation of the construct used for the integration of the copper-sensitive *CTR3* promoter (P_CTR3_). To introduce the promoter into the yeast genome, leucine auxotrophy was complemented by the Kl*LEU2* gene. The construct was flanked by sequences homologous to the *ERG7* promoter (target-up; P_ERG7_) and coding sequence (target-down; *ERG7*) to place the *CTR3* promoter upstream of the endogenous *ERG7* coding sequence. **b** The yeast strain carrying the *CTR3* promoter construct showed a reduction in squalene levels and the accumulation of 2,3-oxidosqualene even without exposure to CuSO_4_ (rox1::P_GAL1_-tHMGR P_GAL10_-ERG13 P_ERG7_Δ::P_CTR3_ 0 μM CuSO_4_). This effect was significantly enhanced in the presence of 150 μM CuSO_4_ (rox1::P_GAL1_-tHMGR P_GAL10_-ERG13 P_ERG7_Δ::P_CTR3_ 150 μM CuSO_4_; *p* = 0.00613 for the reduction of squalene; *p* = 0.00507 for the accumulation of 2,3-oxidosqualene). Higher concentrations of copper (375 μM CuSO_4_) reduced squalene levels and increased the abundance of 2,3-oxidosqualene (rox1::P_GAL1_-tHMGR P_GAL10_-ERG13 P_ERG7_Δ::P_CTR3_ 375 μM CuSO_4_). Standard deviation was calculated from *n* = 3 individual transformants. CDW = cell dry weight; one asterisk = *p* ≤ 0.05; two asterisks = *p* ≤ 0.01; three asterisks = *p* ≤ 0.001

We inserted a 735-bp promoter fragment from the copper transporter gene *CTR3* upstream of the endogenous *ERG7* coding sequence and, in the same step, deleted a 196-bp fragment of the endogenous *ERG7* promoter (Fig. 3a). The inserted *CTR3* promoter fragment contains two *cis*-acting copper responsive elements (TTTGCTC) which inhibit gene expression in the presence of CuSO_4_ (Labbé et al. 1997). A *CTR3* promoter fragment was used successfully to repress *ERG9* expression and thus enhance artemisinin production, thereby lowering the costs of this industrial process, indicating that the *CTR3* promoter is a suitable replacement for the *MET3* promoter that was used in previous studies by the same authors (Paddon et al. 2013).

**Fig. 4:**
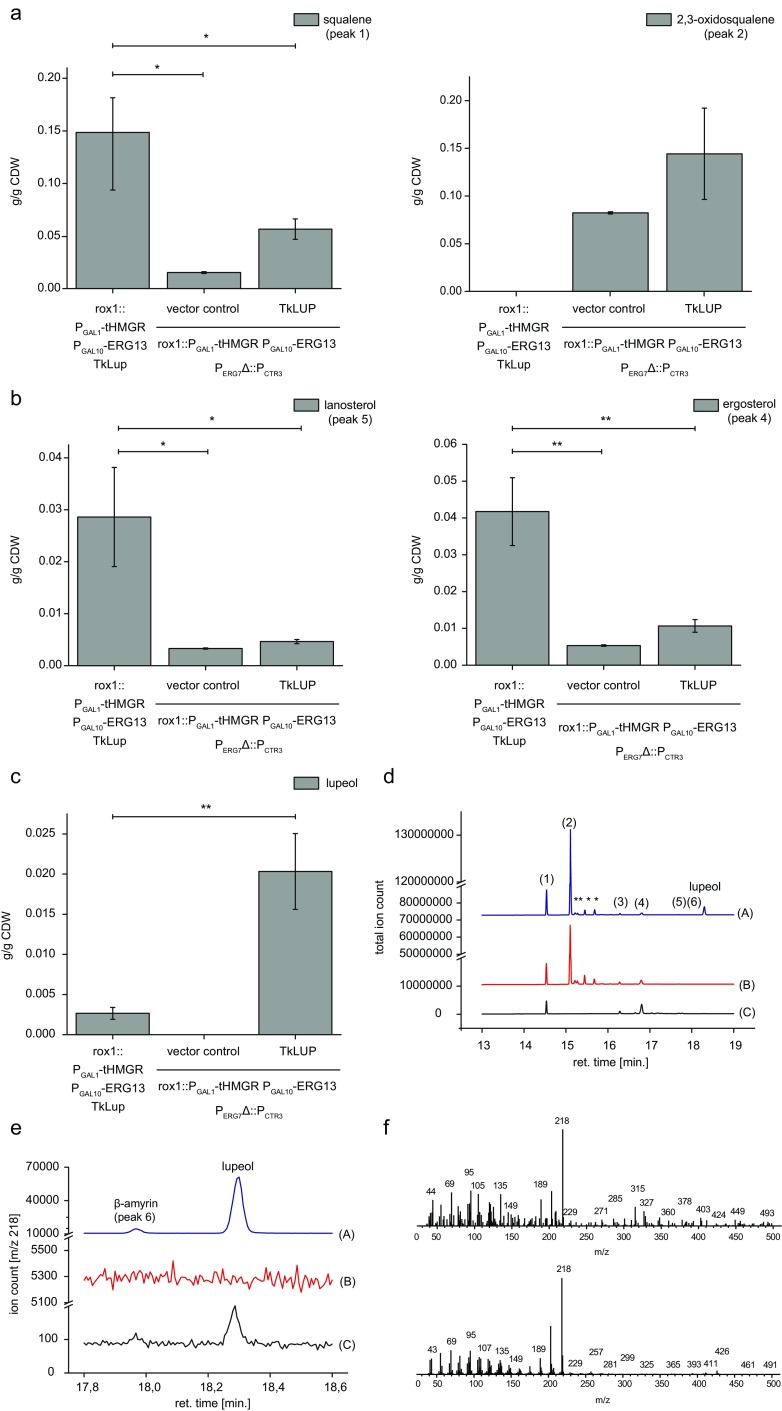
The repression of *ERG7* enhances the accumulation of lupeol while reducing the abundance of sterols. **a** The accumulation of 2,3-oxidosqualene and the lower levels of squalene were observed in the yeast strain carrying the TkLUP sequence in addition to the rox1::P_GAL1_-tHMGR P_GAL10_-ERG13 and the P_ERG7_Δ::P_CTR3_ cassettes, following exposure to 150 μM CuSO_4_ during growth (rox1::P_GAL1_-tHMGR P_GAL10_-ERG13 P_ERG7_Δ::P_CTR3_ TkLUP) compared to the parental strain (rox1::P_GAL1_-tHMGR P_GAL10_-ERG13 TkLUP; *p* = 0.04422 for squalene). **b** The sterol content was reduced as anticipated, as shown by the amounts of lanosterol and ergosterol as representatives for sterol biosynthesis. The repression of *ERG7* resulted in a 6.5-fold decrease in lanosterol levels (*p* = 0.02384) and a 3.9-fold decrease in ergosterol levels (*p* = 0.00941). **c** The repression of sterol biosynthesis enhances lupeol production by 7.6-fold (*p* = 0.00637), suggesting the redirection of the metabolic flux from sterol biosynthesis to lupeol production. **d** Comparison of the total ion count of different yeast strains. (A) = rox1::P_GAL1_-tHMGR P_GAL10_-ERG13 P_ERG7_Δ::P_CTR3_ TkLUP; (B) = rox1::P_GAL1_-tHMGR P_GAL10_-ERG13 P_ERG7_Δ::P_CTR3_ vector control; (C) = TkLUP; (1) = squalene peak; (2) = 2,3-oxidosqualene peak; (3) = cholesterol peak; (4) = ergosterol peak; (5) = lanosterol peak; (6) = β-amyrin peak; asterisk = unidentified yeast metabolites. **e** Detailed comparison of the β-amyrin and lupeol peak [*m*/*z* = 218] from different yeast strains. (A) = rox1::P_GAL1_-tHMGR P_GAL10_-ERG13 P_ERG7_Δ::P_CTR3_ TkLUP; (B) = rox1::P_GAL1_-tHMGR P_GAL10_-ERG13 P_ERG7_Δ::P_CTR3_ vector control; (C) = TkLUP. **f** Identification of β-amyrin in yeast following the repression of *ERG7*. Mass spectrum of the designated β-amyrin peak from the engineered yeast strain (rox1::P_GAL1_-tHMGR P_GAL10_-ERG13 P_ERG7_Δ::P_CTR3_ TkLUP) (upper part). Mass spectrum of the measured external β-amyrin standard (lower part). Standard deviation was calculated from *n* = 3 individual transformants. CDW = cell dry weight; one asterisk = *p* ≤ 0.05; two asterisks = *p* ≤ 0.01

To test the activity of the construct, we introduced the corresponding fragment of pESC_P_ERG7__KlLEU2_P_CTR3__erg7, containing the *CTR3* promoter fragment, into the yeast strain carrying the fragment of pESC-rox1-KlURA3_tHMGR/ERG13. Positive transformants were cultured in the presence of 0, 150, and 375 μM CuSO_4_, and the expression of *tHMGR* and *ERG13* was induced as described in the “Materials and Methods,” resulting in a shift from squalene to 2,3-oxidosqualene accumulation (Fig. 3b). Even in the absence of copper, we detected the accumulation of small amounts of 2,3-oxidosqualene, indicating that the *CTR3* promoter fragment was weaker than the endogenous *ERG7* promoter. This effect was even stronger in the yeast growing in the presence of 150 μM CuSO_4_. The amount of squalene declined significantly (*p* = 0.00613) as the accumulation of 2,3-oxidosqualene increased by 4.7-fold (*p* = 0.00507), which may reflect the absence of *ERG9* and *ERG1* repression by ergosterol, as well as the enhanced induction of *ERG1* due to the lower levels of lanosterol (Table 1) (M’Baya et al. 1989). A further significant decrease in squalene levels (*p* = 0.00061) was observed when the yeast were cultivated in the presence of 375 μM CuSO_4_. However, there was no significant change in 2,3-oxidosqualene levels but the time to reach the cell density for harvesting extended from 20 to 35 h. This indicates that high levels of CuSO_4_ are toxic. We concluded that sufficient repression of *ERG7* was achieved in the presence of 150 μM CuSO_4_, allowing us to overcome the bottleneck in 2,3-oxidosqualene synthesis. We therefore chose 150 μM CuSO_4_ for the enhanced production of lupeol in a strain combining rox1::P_GAL1_-tHMGR P_GAL10_-ERG13 P_ERG7_Δ::P_CTR3_ and the coding sequence of TkLUP, or the pAG424_P_GAL1__*ccdB* empty plasmid serving as a control (Fig. 4).

As expected, the yeast strain carrying all three constructs, when cultivated in the presence of CuSO_4_, showed a shift in squalene and 2,3-oxidosqualene levels compared to its parental strain lacking the *CTR3* promoter fragment. The squalene content of these cells was 2.6-fold lower (*p* = 0.04422), but 2,3-oxidosqualene accumulated instead. Furthermore, the sterol content declined, as shown by the 6.5-fold lower levels of lanosterol (*p* = 0.02384) and 3.9-fold lower levels of ergosterol (*p* = 0.00941), confirming that copper-based repression was sufficient for the regulation of *ERG9* and *ERG1* in the strain expressing *TkLUP*. Moreover, introducing the copper-repressible promoter enhanced the lupeol content by a further 7.6-fold (*p* = 0.00637). No additional peaks representing products of TkLUP, but the already described β-amyrin peak, could be observed in the comparison of the total ion count (Fig. 4d) and the *m*/*z* 218 scan (Fig. 4e). Furthermore, the identification of β-amyrin became possible as shown in the corresponding mass spectra for the sample (Fig. 4f, upper part) and the external β-amyrin standard (Fig. 4f, lower part). The accumulation of 2,3-oxidosqualene and lupeol, as well as the lower levels of sterols, confirmed the redirection of metabolic flux from sterol biosynthesis to lupeol production in this engineered yeast strain.

## Discussion

**Fig. 5:**
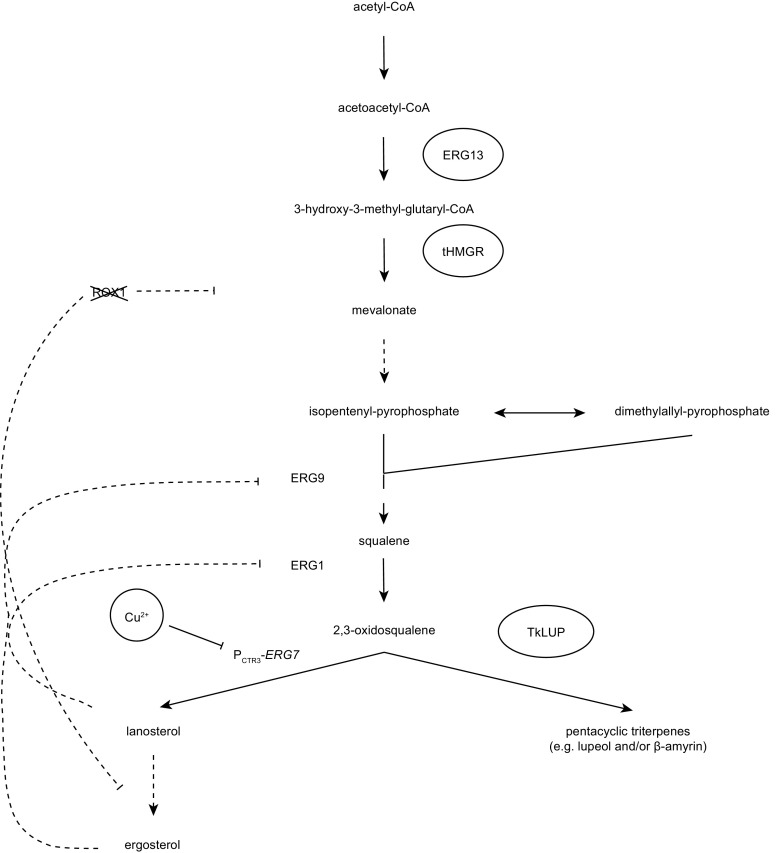
Engineered yeast platform for the enhanced production of pentacyclic triterpenes. The MVA pathway was pushed by the overexpression of *ERG13* and tHMGR, and pulled by the knockout of *ROX1* to enhance the productivity of MVA pathway and late sterol biosynthesis. To redirect metabolic flux from late sterol biosynthesis into the synthesis of pentacyclic triterpenes by the *T. koksaghyz* lupeol synthase (TkLUP), the *CTR3* promoter (P_CTR3_) was used to inhibit the expression of *ERG7* following the addition of copper (Cu^2+^). An additional effect on 2,3-oxidosqualene accumulation was achieved by the missing repression of *ERG9* and *ERG1* due to the lower sterol content (lanosterol and ergosterol). Dashed arrows represent multiple enzymatic reactions

We have established a new platform for the production of pentacyclic triterpenes in yeast, using the lupeol synthase of *T. koksaghyz* as a model enzyme. This platform was based on a push-and-pull strategy combining the overexpression of MVA pathway genes with the deletion of a negative regulator of the MVA pathway and late sterol biosynthesis. Furthermore, we were able to use a copper-regulated promoter to enhance the accumulation of pentacyclic triterpenes by redirecting metabolic flux from late sterol biosynthesis, starting with the formation of lanosterol, to the direct production of the pentacyclic triterpene precursor 2,3-oxidosqualene (Fig. 5).

To accomplish our push-and-pull strategy, we overexpressed the MVA pathway genes *ERG13* (HMGS) and a truncated form of *HMG1* (tHMGR) because others have shown that HMGR is the key rate-limiting step in the pathway and its deregulated form tHMGR can enhance squalene accumulation and isoprenoid levels in yeast platforms (Kirby et al. 2008; Asadollahi et al. 2010; Westfall et al. 2012; Scalcinati et al. 2012; Paddon et al. 2013; Lv et al. 2014; Yuan and Ching 2014). Furthermore, the overexpression of *ERG13* increases flux through the pathway, given that a 13-fold increase in amorpha-4,11-diene production was observed when *ERG13* was overexpressed in combination with *tHMGR*, *ERG10*, and *ERG12* (Yuan and Ching 2014). We chose the *ROX1* locus as an integration site for our overexpression cassette, thus knocking out this negative regulator of the MVA pathway and late sterol biosynthesis (Henry et al. 2002; Montañés et al. 2011; Özaydin et al. 2013; Jakočiūnas et al. 2015). The knockout should upregulate the MVA pathway in general, although the higher levels of squalene resulting from the increased flux were in part consumed by the simultaneously upregulated late sterol biosynthesis pathway. Using this strategy, we were able to increase the accumulation of squalene, lanosterol, ergosterol, and lupeol (Fig. 2b and Table 1). This came at the cost of a slight reduction in growth of our squalene-accumulating yeast strain, consistent with some previous studies (Donald et al. 1997; Asadollahi et al. 2010) but not others (Veen et al. 2003). We did not observe the accumulation of 2,3-oxidosqualene, the direct precursor of pentacyclic triterpene synthesis, so we redirected the flux from late sterol biosynthesis towards the production of pentacyclic triterpenes by replacing the endogenous *ERG7* promoter with the copper-repressible *CTR3* promoter (Labbé et al. 1997). The absence of 2,3-oxidosqualene is therefore likely to reflect the lower capacity of ERG1 compared to ERG9 (Asadollahi et al. 2010) and the rapid conversion of 2,3-oxidosqualene into lanosterol by ERG7 (Veen et al. 2003) or into pentacyclic triterpenes by TkLUP (this study).

As expected, we observed lower squalene levels and the accumulation of 2,3-oxidosqualene following the repression of endogenous *ERG7* in our squalene and pentacyclic triterpene accumulating yeast strain. This may reflect the lower expression of *ERG7* itself, but the regulatory mechanism of *ERG1* and *ERG9* may also contribute to our observations. Because we detected lower levels of ergosterol and lanosterol, the deregulation of a described negative feedback loop may also contribute to the enhanced expression of *ERG9* and *ERG1*. Therefore, the lower sterol content may prevent the suppression of *ERG9* and *ERG1* due to limited ergosterol levels, and may enhance the expression of *ERG1* due to the lack of suppression by lanosterol (M’Baya et al. 1989). By providing the bulk of the direct substrate 2,3-oxidosqualene by the described metabolic engineering steps for the TkLUP enzyme, we were able to enhance the accumulation of lupeol even further and also annotate a thus far uncharacterized peak in the GC-MS spectrum as β-amyrin. In the literature, the product specificity of lupeol synthases ranges from very specific with minor byproducts depending on cultivation conditions (e.g., OEW lupeol synthase from *Olea europaea*, TRW lupeol synthase from *Taraxacum officinale*; Shibuya et al. 1999) to moderate specificity with approximately 20% β-amyrin byproduct (lupeol synthase from *Arabidopsis thaliana*; Herrera et al. 1998). We calculate the β-amyrin byproduct of TkLUP to be approximately 0.6% of total product, classifying it to the higher specificity enzymes.

In conclusion, we were able to enhance the synthesis of pentacyclic triterpenes 127-fold in the case of lupeol within two engineering steps. In addition, we were able to characterize a second pentacyclic triterpene (β-amyrin) synthesized by our model enzyme TkLUP. The unused bulk of 2,3-oxidosqualene could be used as a substrate by other oxidosqualene cyclases to produce even higher amounts of oxidosqualene derivatives. Optimal cultivation techniques could also be used to increase the yields of these molecules (reviewed by Liao et al. 2016).
